# P-570. Need for Broad Protection Against Invasive Meningococcal Disease Among US College Students

**DOI:** 10.1093/ofid/ofaf695.785

**Published:** 2026-01-11

**Authors:** Jessica Presa, Jamie Findlow, Steven Shen, Vincenza Snow, Paul Palmer

**Affiliations:** Pfizer, Inc., Collegeville, PA; Pfizer Ltd, Tadworth, England, United Kingdom; Pfizer Canada ULC, Kirkland, Quebec, Canada; Pfizer Vaccines, Collegeville, PA; Pfizer Vaccine Medical Development, Scientific & Clinical Affairs , Collegeville PA, Collegeville, PA

## Abstract

**Background:**

The Advisory Committee on Immunization Practices (ACIP) recommends a 2-dose meningococcal serogroup B (MenB) vaccination series at age 16–23 years based on shared clinical decision-making (SCDM). Those receiving a MenB vaccine per SCDM who want more rapid protection (eg, students starting vaccination < 6 months before college entry) may receive a 3-dose series. The Meningococcal Work Group of the ACIP proposed several policy options to replace current SCDM recommendations for MenB vaccination with routine or risk-based recommendations. We review epidemiology of invasive meningococcal disease (IMD) and meningococcal vaccination data among college students, who may be included in risk-based recommendations.

**Figure:**
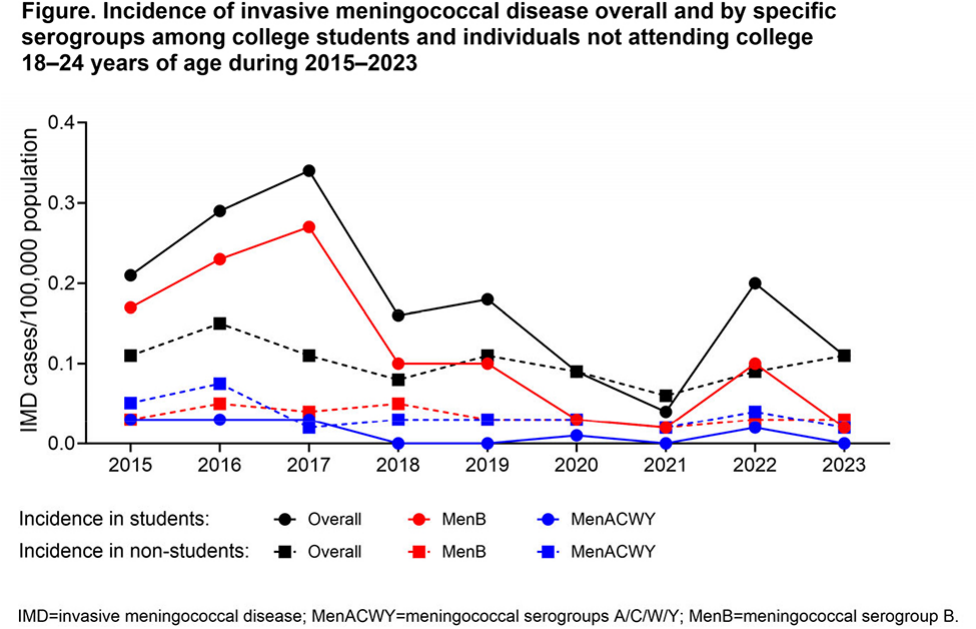

**Methods:**

Data were derived from Enhanced Meningococcal Disease Surveillance reports, the National Notifiable Diseases Surveillance System (NNDSS), and published literature.

**Results:**

Other than a drop during the COVID-19 pandemic (2020–2021), IMD rates during 2015–2023 were similar or higher among 18–24-year-olds attending vs not attending college, with differences dominated by serogroup B (Figure). From 2014–2017 there was a 5.2-fold higher risk of serogroup B IMD among 4-year college students vs those not attending college; risk was increased among those in their first year (3.8-fold), living on campus (2.9-fold), and, during serogroup B outbreaks, participating in Greek life (9.8-fold). NNDSS data show continued increases during 2021–2024 in IMD across all serogroups, with all cases in the US increasing from 208 to 477. Vaccination rates during 2015–2023 among IMD cases for MenACWY and MenB were 76%–100% and 0%–56%, respectively, in college students, and 38%–75% and 0%–18%, respectively, among those not attending college. For the broader population, 2023 vaccination rates among 17-year-olds were 91.2% and 59.7% for ≥ 1 and ≥ 2 MenACWY doses, respectively, and 32.4% and 12.8% for ≥ 1 and ≥2 MenB doses, respectively.

**Conclusion:**

From 2015–2023, compared with non-students, college students had similar or increased incidence of serogroup B IMD. Low vaccination rates among students may be due to the SCDM recommendation for MenB vaccination. Revised meningococcal vaccination strategies should aim for comprehensive protection of this risk group. Funded by Pfizer.

**Disclosures:**

Jessica Presa, MD, Pfizer Inc: Industry|Pfizer Inc: Stocks/Bonds (Public Company) Jamie Findlow, PhD, Pfizer Ltd: Industry|Pfizer Ltd: Stocks/Bonds (Public Company) Steven Shen, MD, PhD, Pfizer Canada ULC: Industry Vincenza Snow, MD, Pfizer Inc: Industry Paul Palmer, PhD, Pfizer Inc: Employee|Pfizer Inc: Stocks/Bonds (Public Company)

